# PHL10/460: Cancerfacts.com - Vertical Portal with Newly Developed Health Profiler

**DOI:** 10.2196/jmir.1.suppl1.e91

**Published:** 1999-09-19

**Authors:** C Lenz, M Brucksch

**Affiliations:** ^1^HNO Universitätsklinik Heidelberg , HeidelbergGermany

**Keywords:** Vertical Portal, Prostate Cancer, Health Profiler

## Abstract

**Introduction:**

Unlike general health portals such as WebMD and Drkoop.com that cover everything from the flu to heart disease, Silicon Valley-based cancerfacts.com is a so-called vertical portal. It covers only one small vertical niche of health care: cancer, and in particular, prostate cancer. As a value-added proprietary technology, the company offers its newly developed profile engine to health information retrievers.

**Methods:**

Users are enabled to insert their specific medical information - results of biopsies, blood tests and exams - into an electronic form, a sort of digital health portfolio.

**Results:**

The software then spits out what treatments are available based on the latest scientific studies. The profiler will even tailor the treatment based on what side effects the user most wants to avoid. Patients can also create "what if" scenarios and find out what clinical trials they can participate in.

**Discussion:**

Cancerfacts.com is riding a trend that is likely to explode in the coming decade. The number one impact the Internet will have on health care, many experts believe, will be empowering individuals to take charge of their own health care. With the profile engine cancerfacts.com has developed a very interesting application which is a new decision-making Internet tool, using technology to create knowledge. Pros and Cons of sites like cancerfacts.com and the future impact of the profile engine are discussed.

